# Gentamicin versus ceftriaxone for the treatment of gonorrhoea (G-TOG trial): study protocol for a randomised trial

**DOI:** 10.1186/s13063-016-1683-8

**Published:** 2016-11-24

**Authors:** Clare Brittain, Margaret Childs, Lelia Duley, Jan Harding, Trish Hepburn, Garry Meakin, Alan A. Montgomery, Wei Tan, Jonathan D. C. Ross

**Affiliations:** 1 Nottingham Clinical Trials Unit, Nottingham Health Science Partners, Queen’s Medical Centre, C Floor South Block, Nottingham, NG7 2UH UK; 2 University Hospitals Birmingham NHS Foundation Trust, Whittall Street Clinic, Whittall Street, Birmingham, B4 6DH UK

**Keywords:** Randomised trial, Gonorrhoea, Gentamicin, Ceftriaxone, Resistance, Treatment, Noninferiority

## Abstract

**Background:**

Gonorrhoea is a common sexually transmitted infection which causes genital pain and discomfort; in women it can also lead to pelvic inflammatory disease and infertility, and in men to epididymo-orchitis. Current treatment is with ceftriaxone, but there is increasing evidence of antimicrobial resistance which is reducing its effectiveness against gonorrhoea. A small, but increasing, number of patients have already been found to have highly resistant strains of gonorrhoea which has been associated with clinical failure. This trial aims to determine whether gentamicin is not clinically worse than ceftriaxone in the treatment of gonorrhoea.

**Methods/design:**

This is a blinded, two-arm, multicentre, noninferiority randomised trial. Patients are eligible if they are aged 16–70 years with a diagnosis of genital, pharyngeal and/or rectal gonorrhoea. Exclusion criteria are: known concurrent sexually transmitted infection(s) (excluding chlamydia); bacterial vaginosis and/or *Trichomonas vaginalis* infection; contraindications or an allergy to gentamicin, ceftriaxone, azithromycin or lidocaine; pregnancy or breastfeeding; complicated gonorrhoeal infection; weight under 40 kg; use of ceftriaxone, gentamicin or azithromycin within the preceding 28 days. Randomisation is to receive a single intramuscular injection of either gentamicin or ceftriaxone, all participants receive 1 g oral azithromycin as standard treatment. The estimated sample size is 720 participants (noninferiority limit 5%).

The primary outcome is clearance of *Neisseria gonorrhoeae* at all infected sites by a negative Nucleic Acid Amplification Test, 2 weeks post treatment. Secondary outcomes include clinical resolution of symptoms, frequency of adverse events, tolerability of therapy, relationship between clinical effectiveness and antibiotic minimum inhibitory concentration for *N. gonorrhoeae*, and cost-effectiveness.

**Discussion:**

The options for future treatment of gonorrhoea are limited. Results from this randomised trial will demonstrate whether gentamicin is not clinically worse than ceftriaxone for the treatment of gonorrhoea. This will inform clinical practice and policy for the treatment of gonorrhoea when current therapy with cephalosporins is no longer effective, or is contraindicated.

**Trial registration:**

International Standard Randomised Controlled Trial Number – ISRCTN51783227, Registered on 18 September 2014. Current protocol version 2.0 17 June 2015.

## Background

Gonorrhoea is the second most common bacterial sexually transmitted infection (STI) in the UK, with 34,958 infections reported in 2014 [1]. A disproportionate burden of infection is seen in young adults and minority ethnic groups (35% of infections). The highest rates of infection are in large urban areas, and infection is concentrated in core high-risk groups such as men who have sex with men (MSM), black and minority ethnic (BME) groups, and those reporting multiple sexual partners. There has recently been a significant rise in rectal gonorrhoea in MSM thought to reflect an increase in unsafe sexual behaviour [2].

Gonorrhoea leads to local inflammation causing genital pain and discomfort, and the localised immune activation also facilitates the acquisition and transmission of HIV. In women, infection can spread to the fallopian tubes and ovaries causing pelvic inflammatory disease with resultant tubal scarring, infertility, chronic pelvic pain and an increased risk of ectopic pregnancy. In men, gonorrhoea can lead to epididymo-orchitis and, in men who have sex with men, gonococcal proctitis can lead to abscess and fistula formation. Pharyngeal infection, whilst usually asymptomatic, is an important reservoir of onward transmission in both women and MSM. It is also harder to treat with antibiotics [3] and can persist even when sensitivity testing suggests that it should be susceptible [3]. It is, therefore, important to find out whether treatment is effective for infection at all anatomical sites.

### Antibiotic treatment and resistance

*Neisseria gonorrhoeae* readily develops resistance to antibiotic regimens. There are now high levels of resistance against penicillins, sulphonamides, tetracyclines and quinolones, all of which are no longer recommended for use. A real possibility of multidrug-resistant gonorrhoea and the lack of treatment options has recently been highlighted [4]. Guidance from the British Association for Sexual Health and HIV (BASHH) is to treat with ceftriaxone (given with adjunctive azithromycin) and this currently cures over 95% of patients [2]. Recent surveillance data show a reduction in sensitivity to ceftriaxone with an upward drift in the minimum inhibitory concentration (MIC) (13% with MIC over 0.03 mg/l in 2010 cf. 1% in 2007), i.e. the proportion of cases which remain highly sensitive to ceftriaxone has decreased over time [5]. Sporadic clinical failure of cephalosporins has been reported from Japan, Sweden, Spain and France [6–10]. The same reduction in antibiotic sensitivity was followed by widespread clinical failure within a few years for other antimicrobials (penicillin, tetracyclines and quinolones) used to treat gonorrhoea. More recently an outbreak of azithromycin-resistant gonorrhoea has been reported in England further highlighting the need to identify other effective treatment regimens [11]. Despite this recent outbreak azithromycin resistance remains uncommon in England and levels of azithromycin resistance have not increased over the past 18 months since the outbreak was first identified. Current national and international treatment guidelines continue to recommend dual antibiotic therapy, including azithromycin, for the treatment of gonorrhoea. The ongoing use of azithromycin will be monitored during the trial to ensure that its use remains appropriate.

### Alternative treatment

If cephalosporins become ineffective the options for treating gonorrhoea are currently limited. With the exception of gentamicin, alternative agents have either not been assessed in vivo (such as ertapenem, solithromycin) [12–15], are reserved for specific infections (e.g. rifampicin for tuberculosis) [16] or have the potential to rapidly develop resistance (e.g. spectinomycin) [17].

Two recent systematic reviews on gentamicin monotherapy reported cure rates for gentamicin of 62–98% in patients with gonorrhoea, but available studies were generally small and of low quality with a significant risk of bias and random error [18, 19]. No adverse events (AEs) were reported in these studies. A more recent two-arm prospective study evaluated single-dose gentamicin combined with 2 g oral azithromycin with a reported cure rate of 100% [20]. Limited data are available on the efficacy of gentamicin when treating gonorrhoea in the pharynx or rectum, although antibiotics are sometimes less effective at these sites.

### Why a trial is needed now

As the susceptibility of *N. gonorrhoeae* to currently recommended antibiotics decreases and multidrug-resistant strains become more common, it is important to demonstrate the efficacy and safety of alternative treatment regimens in patients with gonorrhoea. Gentamicin is a cheap and widely available antibiotic. It has been associated with renal and vestibulo-cochlear toxicity, which is partially dose-related [21], and the use of a single one-off dose appears to be well-tolerated [22]. In vitro microbial sensitivity data [23] support the use of gentamicin but there is need for clinical trial data to assess its efficacy and safety, particularly in pharyngeal and rectal infections.

Our hypothesis is that gentamicin is not clinically worse than ceftriaxone in the treatment of gonorrhoea. The aim is to test this hypothesis in a randomised trial by comparing microbiological clearance of *N. gonorrhoeae* following treatment with gentamicin or with ceftriaxone.

## Methods/design

This is a blinded, two-arm, multicentre, noninferiority, randomised trial comparing the clinical effectiveness and safety of gentamicin and ceftriaxone in the treatment of gonorrhoea.

### Participants

Patients are eligible for the trial if they are aged 16–70 years and have received a positive diagnosis in the last 4 weeks of uncomplicated, untreated (not received any antibiotic in the previous 28 days which could have treated gonorrhoea, either partially or completely) genital, pharyngeal and/or rectal gonorrhoea. Diagnosis must be based on a positive gram-stained smear on microscopy, or a positive Nucleic Acid Amplification Test (NAAT). Current treatment guidelines do not differentiate their recommendations by sex of patient and there is no evidence from previous studies that treatment response differs between men and women, thus both male and female patients are eligible to take part in the trial.

Exclusion criteria are known concurrent bacterial STI (apart from chlamydia); known bacterial vaginosis and/or *Trichomonas vaginalis* infection; known contraindications or an allergy to gentamicin, ceftriaxone, azithromycin or lidocaine; patients with a current clinical diagnosis of complicated gonorrhoeal infections, for example pelvic inflammatory disease, epididymo-orchitis; patients who weigh less than 40 kg; patients who are receiving or have received ceftriaxone, gentamicin or azithromycin within the preceding 28 days. Pregnant and breastfeeding women are also excluded. Patients are only eligible to participate in the trial once.

Patients are recruited from 13 outpatient sexual health clinics in England, all of which offer direct self-referral with both a walk-in service and prebookable appointments. Clinics are situated in urban locations and care is provided by specialist medical and nursing staff following national management guidelines [24]. Some clinics seeing a high proportion of MSM were purposely chosen to maximise the number individuals with pharyngeal and rectal infections included in the trial.

### Interventions

Participants are randomised to receive either gentamicin 240 mg (intervention) or ceftriaxone 500 mg (current standard treatment) and treatment is administered after obtaining consent during the same clinic visit.

Both treatments are administered from routine clinic stock as a single intramuscular injection. Ceftriaxone (500 mg) is a powder formulation and is dissolved in 1% lidocaine in accordance with the Summary of Product Characteristics (SmPC) and administered as a single 2-ml intramuscular injection. Gentamicin (240 mg) is made up from 3 × 2-ml (80-mg) vials in accordance with the SmPC and administered as a single 6-ml intramuscular injection. In addition, all participants receive a single oral dose of 1 g azithromycin, which is currently given as standard treatment alongside ceftriaxone.

Previous trials have most commonly used a 240-mg dose of gentamicin and the use of different doses has not demonstrated a significant dose-response effect across studies. In vitro susceptibility testing also suggests that isolates remain sensitive to gentamicin [23, 25]. The dose of ceftriaxone was chosen to be consistent with current UK gonorrhoea treatment guidelines [24].

### Outcomes measures

The primary outcome measure is clearance of *N. gonorrhoeae* at all infected sites confirmed by a negative NAAT 2 weeks post treatment (as recommended by the British Association for Sexual Health and HIV).

The NAAT is an automated laboratory test and, therefore, it is not subject to bias through knowledge of treatment allocation. The method of NAAT (e.g. Aptima Combo (AC), Becton Dickinson (BD), Roche Cobas) performed varies between sexual health centres; therefore, in order to ensure consistency and standardisation in diagnostic and follow-up tests performed by local laboratories, additional samples are taken from participants recruited at centres where the AC NAAT method is not used by the local laboratory. Testing of these samples by AC NAAT is performed by a central laboratory (the Sexually Transmitted Bacteria Reference Unit (STBRU) within Public Health England). The results from the AC NAAT will be used to assess clearance for the primary endpoint.

Secondary outcomes are:

- Clinical resolution of symptoms
- frequency of nausea/vomiting, hearing loss, dizziness and rash
- Frequency of any other AEs reported by participants
- tolerability of injection as assessed by the participant on a Visual Analogue Scale (VAS)
- Cost-effectiveness

The relationship between clearance of gonorrhoea and in vitro measurement of antibiotic MIC to inhibit *N. gonorrhoeae* growth will be assessed.

Effectiveness, tolerability and safety are assessed at a follow-up visit 2 weeks post treatment.

### Sample size

Based on a cure rate of 96% for the ceftriaxone regimen, which is consistent with previous trials, a total sample size of 646 for analysis (323 in each group) will achieve a 90% power to detect noninferiority with lower confidence interval for an absolute risk difference of 5%. The one-sided significance level is 0.025. To allow for a loss to follow-up rate of up to 10%, the trial will recruit a total of 720 participants.

### Consent

Patients with a provisional (indicated by a positive gram stain of genital secretions on microscopy) or confirmed (indicated by a positive NAAT) diagnosis of gonorrhoea are screened for the trial and approached by a member of the site research team to determine whether they are interested in participating. They are provided with a Patient Information Sheet and given a verbal explanation of the trial with the opportunity to ask any questions and have these addressed. In addition, trial posters are on display in relevant areas of the clinic. These help to introduce the trial and, if patients are interested, they can ask clinic staff for additional details.

To avoid delaying treatment for a transmissible infection with serious sequelae, patients with either a provisional (on microscopy) or confirmed diagnosis (on NAAT) of untreated gonorrhoea are invited to participate and provide written consent at the same clinic visit.

### Randomisation

After providing consent, participants are registered in the trial using a web-based registration and randomisation system. Once eligibility is confirmed, participants are randomised to either gentamicin or ceftriaxone by a member of the research team. Staff who perform randomisation have no role in administering trial treatments and remain blinded to the treatment allocation, thereby minimising risk of selection bias through prediction of the allocation sequence.

Randomisation is based on a computer-generated pseudo-random code using permuted blocks of randomly varying size, created by the Nottingham Clinical Trials Unit (NCTU) in accordance with their Standard Operating Procedure and held on a secure server. Randomisation is in a 1:1 ratio, stratified by recruiting centre.

The web-based system generates a blinded prescription for G-TOG trial treatment which must be signed by the prescribing doctor. Site staff record only ‘G-TOG study drug’ in the participant’s medical notes. The signed prescription is passed to a nurse who administers the allocated treatment.

### Blinding of the assessment of outcome

Nurses administering trial treatments are required to know each participant’s allocation since they must formulate the drug into a single injection. Details of the nurses administering treatment at each centre are obtained during the trial set-up stage and access to the online randomisation allocation is granted according to the role. All other staff at the recruiting centres remain blinded to treatment allocation.

Preparation and administration of trial treatments is undertaken in a separate area away from the blinded research team and the participant. In addition, nurses administering treatment are given guidance to provide standardised information to participants at the time of injection, which is the same regardless of treatment allocation, to prevent inadvertent unblinding. This two-step approach maintains blinding for members of the research team who are subsequently involved in the assessment of the participant.

To ensure that assessment of outcome is not influenced by knowledge of the allocated treatment, nurses administering trial treatments are not permitted any role in the collection of outcome data to reduce risk of ascertainment bias.

### Baseline visit

During the baseline visit, demographic information is collected together with details of the participant’s sexual history and their symptoms.

For symptomatic participants, the baseline visit usually takes place on the same day as diagnosis based on the appearances of gram-stained genital discharge on microscopy. Asymptomatic participants are recalled to clinic for treatment following a positive diagnosis; once they have given consent to participate in the trial this second visit (the first following diagnosis) is considered as the baseline visit.

Each participant has swabs taken for NAAT and culture testing to determine the sites of infection. A full sampling profile is required according to the participant’s gender and sexual orientation to reflect potential sites of exposure. This will allow the efficacy of treatment to be assessed at each infected site. Where swabs are not part of routine clinical care or have not been taken already during the baseline clinic visit (e.g. symptomatic participants where only swabs have been taken for routine care on the same day prior to consent), additional swabs are taken to complete the full sampling profile as defined in Table 1.

**Table 1:** Sampling schedule for trial participants

Sex/reported sexual orientation	Genital sample	Pharynx	Rectum
Women	✔^a^	✔^c^	✔^c^
Heterosexual men	✔^b^	X	X
Men who have sex with men	✔^b^	✔^c^	✔^c^

^a^Culture sample from cervix, Nucleic Acid Amplification Test (NAAT) sample from vagina or cervix

^b^Culture sample from urethra, NAAT sample from urine or urethra

^c^Culture sample plus NAAT sample

All samples collected are sent to local site laboratories for analysis and results are reported back to the clinic in the usual manner. The results from the baseline visit inform subsequent testing of previously infected sites at the follow-up visit.

In centres where AC NAAT is not used for local testing, an additional set of swabs is taken for AC NAAT by the central laboratory. The results of any tests performed by the central laboratory are not provided back to the clinic and are reported in batches to the NCTU. Management of the participant is based only on the results of local testing.

An additional blood sample is taken for measurement of the pretreatment immune response to gonococcal infection.

### Follow-up of participants

**Fig. 1:**
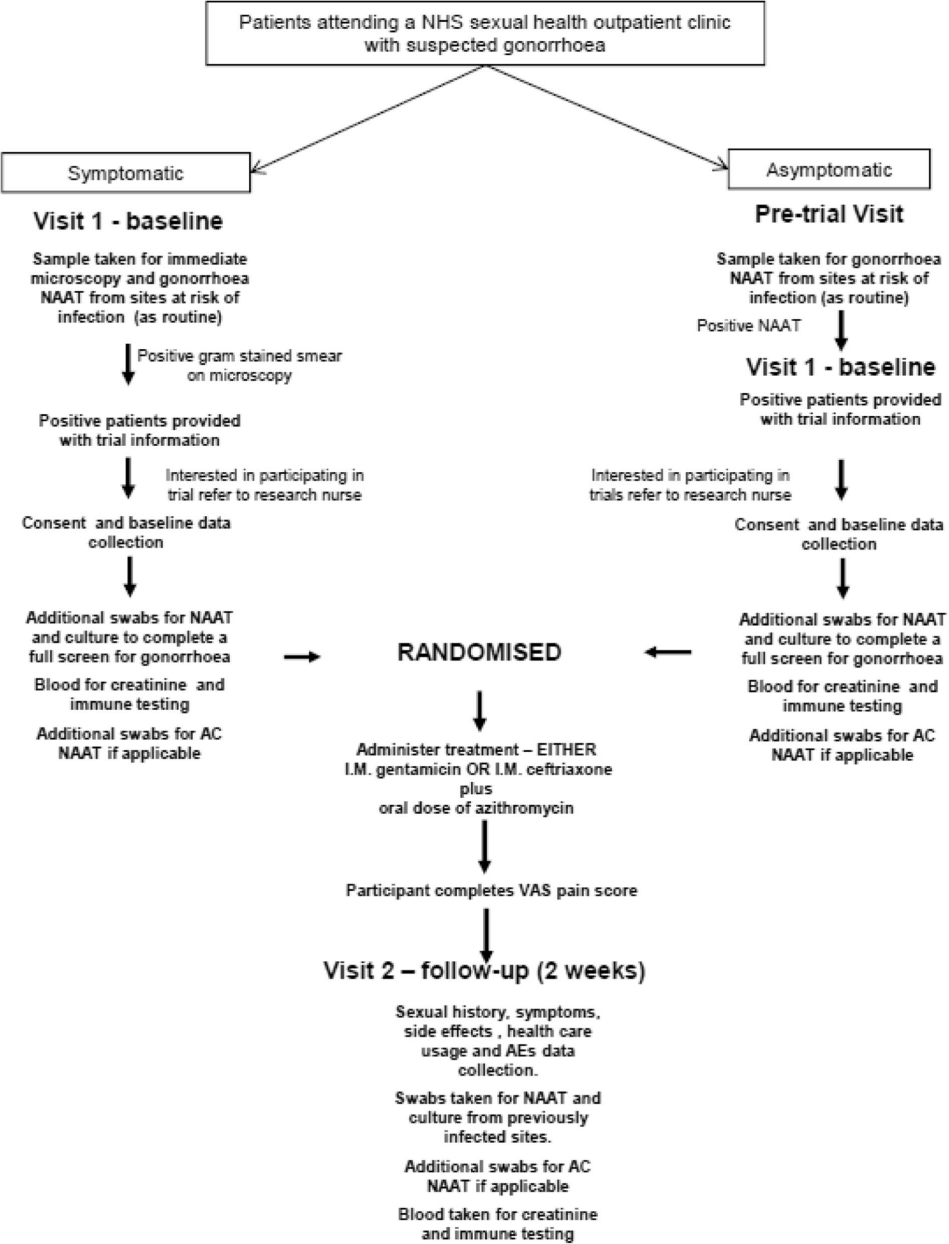
Trial flow diagram

Participants are asked to return to clinic 2 weeks post treatment (which is also 2 weeks post randomisation) for a follow-up visit. Participants are reminded of their appointments using the individual clinic’s existing recall procedures such as by short messaging service (SMS) messaging and telephone reminders. During the follow-up visit swabs from previously infected sites are taken for NAAT and culture to assess clearance of *N. gonorrhoeae.* A blood sample is taken to measure the post-treatment immune response. Each participant remains in the trial until this follow-up visit is completed. Participants are considered lost to follow-up if they have not returned for their follow-up appointment within 60 days of the baseline visit; this time point was chosen to allow younger participants, who are often working or have childcare commitments, enough flexibility to return to clinic, whilst not leaving it so long that the risk of reinfection was increased. The recruitment flow diagram is shown in Fig. 1.

If follow-up test results show that the participant has gonorrhoea, they are offered further investigation and treatment according to local clinic guidelines. This is not considered as part of the trial.

### Clinical resolution of symptoms

Participants are asked about their symptoms at the baseline visit, and they may also have a clinical examination if clinically indicated. Resolution of symptoms is assessed at the follow-up visit 2 weeks later.

### Frequency of adverse events and tolerability of therapy

Following the administration of the intramuscular treatment (baseline visit), participants are asked to complete a VAS to assess any pain associated with their injection. Participants are then asked to complete a second VAS at the follow-up visit to again assess their perception of injection discomfort experienced at baseline.

Adverse events and serious AEs from baseline to the end of the follow-up visit are collected. The known side effects of both gentamicin and ceftriaxone (nausea/vomiting, hearing loss, dizziness and rash) are assessed by direct questioning during the follow-up visit by a member of the research team blind to the allocated treatment group. Ototoxicity and nephrotoxicity are the most common side effects associated with gentamicin therapy. Both effects are related to renal impairment and their frequency following a single dose of gentamicin is not known. Other AEs are collected by direct questioning of the participant by a member of the research team at site; participants are asked about any additional new symptoms or complaints (i.e. those not existing before administration of the treatment). In addition, all participants have a blood sample taken at the baseline and follow-up visit to assess changes in creatinine level.

### Cost-effectiveness

Participants are asked questions during their follow-up visit about any additional health resource (e.g. GP or other clinic visit) use since the baseline visit. The economic analysis will compare the costs associated with the current standard treatment, ceftriaxone, with those of the proposed alternative treatment, gentamicin, in the treatment of gonorrhoea. Given that the primary objective of the trial is to determine noninferiority of gentamicin compared to ceftriaxone, the economic analysis will focus on establishing whether the use of gentamicin rather than ceftriaxone is cost-neutral in the treatment of gonorrhoea. This will involve the examination of costs and resource use to determine whether there are any differences between the two treatments.

### Data management and trial monitoring

Data are entered into a trial-specific database developed and maintained by the Nottingham Clinical Trials Unit (NCTU). Access to the database is restricted and secure. All trial data are anonymised by use of unique participant trial numbers. Data quality is checked using criteria for out-of-range and consistency, and checks for conflicting data within and between Data Collections Forms. Missing data and data queries are referred promptly back to the recruiting site for clarification.

Trial monitoring is by central statistical monitoring combined with site visits. Central statistical monitoring is used to assess compliance with protocol, monitor patterns of recruitment at sites, reasons for nonrecruitment and tracking of participant samples. Each site will have at least one monitoring visit. The timing of this visit is influenced by recruitment, data quality and compliance with the protocol and trial procedures.

The trial is conducted in accordance with the ethical principles that have their origin in the Declaration of Helsinki, 2013, with relevant regulations and with MRC Guidelines for Good Clinical Practice in Clinical Trials which is based on International Conference on Harmonisation (ICH) guidelines for Good Clinical Practice (GCP) (CPMP/ICH/135/95) July 1996.

### Statistical analyses

Demographic and clinical measures will be compared between the randomised arms at baseline using appropriate descriptive statistics for continuous and categorical variables.

The primary approach to between-group comparative analyses will be by intention-to-treat without imputation of missing outcome data. Sensitivity analyses will be conducted to investigate the impact of missing primary outcome data, using simple and multiple imputation. Due to both interventions being single-dose administered immediately following randomisation, nonadherence with treatment allocation is expected to be low. Therefore, additional sensitivity analyses that aim to estimate treatment effect amongst those who have adhered with allocation are not expected to be required.

The evaluation of the primary clinical outcome variable will be performed using a general linear model for binary outcome adjusted by clinic site. The primary efficacy parameter comparing gentamicin with ceftriaxone will be the difference in the proportion of participants clear of infection at 2 weeks follow-up along with the 95% confidence interval. Gentamicin will be regarded as noninferior if the lower 95% confidence limit for the risk difference in confirmed clearance is −5 percentage points or greater.

Secondary outcomes will be similarly analysed using appropriate regression models dependent on data type, adjusted for clinic site and baseline value of the outcome variable if collected. These analyses will be considered supportive to the primary analyses.

To explore treatment efficacy by site of infection, for each of the three infection sites, we will separately estimate clearance by treatment arm along with 95% confidence intervals, rather than formally fit an interaction term for the seven different possible combinations of infection site in the regression model. Any suggestion of a differential effect according to infected site would require confirmation in future research.

The relationship between clinical effectiveness and MIC will be examined visually.

Safety and tolerability analyses will be descriptive. Frequency counts and percentages of the prespecified main categories of AEs will be presented by treatment arm.

All planned analyses will be described in the Statistical Analysis Plan which will be finalised prior to database lock and unblinding of the trial.

### Trial management and oversight

Day-to-day management of the trial is the responsibility of the Trial Management Group (TMG), which meets at least every 2 months and more often if required. Trial oversight is by an independent Trial Steering Committee (TSC). Safety of trial participants is monitored by an independent Data Monitoring Committee (DMC), which reports to the TSC. The trial is coordinated by the NCTU.

## Discussion

The G-TOG trial is designed to be a pragmatic trial which aims to reflect routine NHS clinical practice and thus trial procedures, as far as possible, mirror routine clinical practice. Careful consideration and training was given during the set-up stage of the trial to ensure that maintenance of the blind was both feasible and practicable at each participating site without causing any additional burden of work for site staff. An initial assessment of current practice was conducted to identify any potential problems at each site in concealing treatment allocation from both participant and the blinded site research team. This included suitability of the areas where treatment is administered to ensure adequate privacy from any blinded members of the research team. Nurses administering treatment are trained on the importance of provision of consistent information to all participants, regardless of treatment allocation. Treatment administered to trial participants is obtained from standard clinic stock, thus it was also essential to consider whether access to the storage areas for drugs could also result in inadvertent unblinding.

A key challenge for site staff in recruiting patients into the trial is the need to ensure adequate availability of GCP and trial-trained staff since the trial demands both a blinded research nurse/physician and an unblinded nurse administering treatment to perform separate roles during the initial clinic visit. This has meant training a larger number of staff than would ordinarily be required for a trial of this size.

The trial is designed so that participants, clinicians and researchers are blinded to treatment allocation. Whilst the primary end point of the trial is objective and so not subject to bias from knowledge of treatment intervention, blinding to allocation is necessary to minimise selection bias at recruitment and to ensure that ascertainment of outcome and assessment of safety is not biased by knowledge of the administered treatment.

Gentamicin is not currently recommended for use in the UK to treat gonorrhoea due to a lack of efficacy and safety data. The results of this trial will assess whether gentamicin is not clinically worse than ceftriaxone for the treatment of gonorrhoea, when resistance to current antibiotics leads to clinical failure. The trial will generate knowledge about the efficacy of gentamicin in the treatment of gonorrhoea, both at genital and nongenital sites, extend our knowledge of the safety of single-dose gentamicin therapy and provide data to correlate laboratory-based sensitivity testing with the clinical response to treatment (thus helping to calculate the MIC ‘breakpoints’ which can be used to predict from laboratory testing whether a patient will respond to treatment).

It is expected that the results of the trial will inform future guidelines on the treatment of gonorrhoea, influence policy recommendations on antimicrobial testing and surveillance, and assist developing countries in choosing evidence-based affordable gonorrhoea treatment.

## Trial status

Recruitment to the G-TOG trial commenced in October 2014 and is expected to continue until the end of October 2016. So far, 593 patients have been randomised (as of 11 July 2016). At the time of writing, thirteen sexual health outpatient clinics in England were participating in the trial.

## Abbreviations

AC: Aptima Combo
AE: Adverse event
BASHH: British Association for Sexual Health and HIV
BD: Becton Dickinson
CI: Chief investigator
DMC: Data Monitoring Committee
GCP: Good Clinical Practice
MIC: Minimum inhibitory concentration
MSM: Men who have sex with men
NAAT: Nucleic Acid Amplification Test
NCTU: Nottingham Clinical Trials Unit
STBRU: Sexually Transmitted Bacteria Reference Unit
STI: Sexually transmitted infection
TSC: Trial Steering Committee
UHBFT: University Hospitals Birmingham NHS Foundation Trust
VAS: Visual Analogue Scale

## References

[1] Public Health England. Sexually transmitted infections and chlamydia screening in England, 2014. Health Protection Report 2015. https://www.gov.uk/government/statistics/sexually-transmitted-infections-stis-annual-data-tables

[2] Public Health England. Surveillance of antimicrobial resistance in *Neisseria gonorrhoeae*. Key findings from the ‘Gonococcal Resistance to Antimicrobials Surveillance Programme’ (GRASP) and related surveillance data. 2014. https://www.gov.uk/government/uploads/system/uploads/attachment_data/file/476582/GRASP_2014_report_final_111115.pdf.

[3] Moran JS, Levine WC (1995). Drugs of choice for the treatment of uncomplicated gonococcal infections. Clin Infect Dis..

[4] Duncan S, Duncan CJ (2012). The emerging threat of untreatable gonococcal infection. N Engl J Med.

[5] Group GRASP. GRASP 2010 Report: The Gonococcal Resistance to Antimicrobials Surveillance Programme. Public Health England. 2011. http://webarchive.nationalarchives.gov.uk/20140714084352/.

[6] Unemo M, Golparian D, Nicholas R, Ohnishi M, Gallay A, Sednaoui P (2012). High-level cefixime- and ceftriaxone-resistant *Neisseria gonorrhoeae* in France: novel penA mosaic allele in a successful international clone causes treatment failure. Antimicrob Agents Chemother.

[7] Unemo M, Golparian D, Hestner A. Ceftriaxone treatment failure of pharyngeal gonorrhoea verified by international recommendations, Sweden, July 2010. Euro Surveill. 2011;16(6). 21329645

[8] Ison CA, Hussey J, Sankar KN, Evans J, Alexander S. Gonorrhoea treatment failures to cefixime and azithromycin in England, 2010. Euro Surveill. 2011;16(14). 21492528

[9] Ohnishi M, Golparian D, Shimuta K (2011). Is *Neisseria gonorrhoeae* initiating a future era of untreatable gonorrhea?: detailed characterization of the first strain with high-level resistance to ceftriaxone. Antimicrob Agents Chemother.

[10] Forsyth S, Penney P, Rooney G. Cefixime-resistant *Neisseria gonorrhoeae* in the UK: a time to reflect on practice and recommendations. Int J STD AIDS. 2011;22(5):296–7. 10.1258/ijsa.2009.009191 21571983

[11] Public Health England. Health Protection Report. 2016;10(15). https://www.gov.uk/government/uploads/system/uploads/attachment_data/file/517644/hpr1516.pdf.

[12] Livermore DM, Alexander S, Marsden B (2004). Activity of ertapenem against *Neisseria gonorrhoeae*. J Antimicrob Chemother.

[13] Olsen B, Pham TL, Golparian D, Johansson E, Tran HK, Unemo M (2013). Antimicrobial susceptibility and genetic characteristics of *Neisseria gonorrhoeae* isolates from Vietnam, 2011. BMC Infect Dis..

[14] Golparian D FP, Ohnishi M, Jensen JS, Unemo M. In vitro activity of solithromycin (CEM-101) against clinical *Neisseria gonorrhoeae* isolates displaying various types of antimicrobial resistance profiles. Clinical Microbiology and Infection Conference: 22nd European Congress of Clinical Microbiology and Infectious Diseases London United Kingdom Conference Start: 20120331 Conference End: 20120403. Conference Publication: (var pagings). 2012;18:391.

[15] Unemo M, Golparian D, Limnios A (2012). In vitro activity of ertapenem versus ceftriaxone against *Neisseria gonorrhoeae* isolates with highly diverse ceftriaxone MIC values and effects of ceftriaxone resistance determinants: ertapenem for treatment of gonorrhea?. Antimicrob Agents Chemother.

[16] Panduro J (1971). Treatment of acute gonorrhoea with a single oral dose of rifampicin. Br J Vener Dis.

[17] Boslego JW, Tramont EC, Takafuji ET (1987). Effect of spectinomycin use on the prevalence of spectinomycin-resistant and of penicillinase-producing *Neisseria gonorrhoeae*. N Engl J Med.

[18] Dowell D, Kirkcaldy RD (2012). Effectiveness of gentamicin for gonorrhoea treatment: systematic review and meta-analysis. Sex Transm Infect.

[19] Hathorn E, Dhasmana D, Duley L, Ross JD (2014). The effectiveness of gentamicin in the treatment of *Neisseria gonorrhoeae*: a systematic review. Syst Rev..

[20] Kirkcaldy RD, Weinstock HS, Moore PC (2014). The efficacy and safety of gentamicin plus azithromycin and gemifloxacin plus azithromycin as treatment of uncomplicated gonorrhea. Clin Infect Dis.

[21] British National Formulary. https://www.bnf.org/.

[22] Hayward SR, Harding J, Molloy R, Land L, Longcroft-Neal K, Moore D, Ross JDC. Adverse effects of single dose gentamicin in adults: a systematic review. Oral Presentation IUSTI World Confree 2016, Marrakesh, Morocco. 2016.

[23] Chisholm SA, Quaye N, Cole MJ (2011). An evaluation of gentamicin susceptibility of *Neisseria gonorrhoeae* isolates in Europe. J Antimicrob Chemother.

[24] British Association for Sexual Health and HIV (BASHH) Clinical Effectiveness Group Guidelines. https://www.bashh.org/guidelines.

[25] Brown LB, Krysiak R, Kamanga G, et al. *Neisseria gonorrhoeae* antimicrobial susceptibility in Lilongwe, Malawi, 2007. Sex Transm Dis. 2010;37(3):169–72. 10.1097/OLQ.0b013e3181bf575c 19901860

